# Catch-up growth is a better indicator of undernutrition than thresholds for stunting

**DOI:** 10.1017/S1368980020003067

**Published:** 2021-01

**Authors:** Christiane Scheffler, Barry Bogin, Michael Hermanussen

**Affiliations:** 1 Institute of Biology and Biochemistry, Human Biology, University of Potsdam, Am Neuen Palais 10, 14467 Potsdam, Germany; 2 School of Sport, Exercise & Health Sciences, Loughborough University, Loughborough, UK; 3 Christian-Albrecht-Universität Kiel, Aschauhof 3, Eckernförde – Altenhof, Germany

**Keywords:** No threshold for stunting, Catch-up growth, Social-economic-political-emotional (SEPE) factors

## Abstract

**Objective:**

Stunting (height-for-age < −2 sd) is one of the forms of undernutrition and is frequent among children of low- and middle-income countries. But stunting *per* *s* *e* is not a synonym of undernutrition. We investigated association between body height and indicators of energetic undernutrition at three critical thresholds for thinness used in public health: (1) BMI SDS < −2; (2) mid-upper arm circumference divided by height (MUAC (mm) × 10/height (cm) < 1·36) and (3) mean skinfold thickness (SF) < 7 mm and to question the reliability of thresholds as indicators of undernutrition.

**Design:**

Cross-sectional study; breakpoint analysis.

**Setting:**

Rural and urban regions of Indonesia and Guatemala – different socio-economic status (SES).

**Participants:**

1716 Indonesian children (6·0–13·2 years) and 3838 Guatemalan children (4·0–18·9 years) with up to 50 % stunted children.

**Results:**

When separating the regression of BMI, MUAC or SF, on height into distinguishable segments (breakpoint analysis), we failed to detect relevant associations between height, and BMI, MUAC or SF, even in the thinnest and shortest children. For BMI and SF, the breakpoint analysis either failed to reach statistical significance or distinguished at breakpoints above critical thresholds. For MUAC, the breakpoint analysis yielded negative associations between MUAC/h and height in thin individuals. Only in high SES Guatemalan children, SF and height appeared mildly associated with *R* ^2^ = 0·017.

**Conclusions:**

Currently used lower thresholds of height-for-age (stunting) do not show relevant associations with anthropometric indicators of energetic undernutrition. We recommend using the catch-up growth spurt during early re-feeding instead as immediate and sensitive indicator of past undernourishment. We discuss the primacy of education and social-economic-political-emotional circumstances as responsible factors for stunting.

Undernutrition impairs growth. Multiple sources of historic evidence of child hunger during and after World War I and World War II were summarised by Keys *et al*.^(1)^. More recent historic literature refers to the work of Gomez and colleagues^(2)^ who distinguished between first-degree malnutrition with ‘actual body weight between 85 and 75 % of the average theoretic weight for age’; second-degree malnutrition,* with ‘actual body weight between 75 and 60 % of the average theoretic weight for age’ and third-degree malnutrition, with ‘actual body weight <60 % of the average theoretic weight for age’. Gomez and colleagues studied third-degree malnutrition in 584 children aged 6 months to 5 years, referred to the Hospital Infantil de Mexico, in Mexico City. These children exhibited classic clinical symptoms of severe food shortage, and they were short. On average, they reached 84·2 % of their theoretic height for age. This study was repeatedly cited in the subsequent decades. When in the early 1970s, Waterlow classified protein–energy malnutrition and introduced the term ‘stunting’^(3,4)^, he referred to the Gomez *et al*. article as justification for choosing 85 % of the average theoretic height-for-age as the cut-off for stunting. A similar cut-off was also chosen by Pelletier^(5)^ when discussing the relationship between child mortality and height-for-age. Death rates per 1000 children rose markedly at average population height below 85 % of theoretic height-for-age. Both Gomez *et al*. and Pelletier referred to average population height, but not to individual children. In their papers, it remained unclear whether a particular child below the 15 % deficit in height showed more, or more severe, clinical signs of undernutrition, or was at greater risk of death than children above this cut-off.

The historic phrase ‘percent of theoretic height for age’ has been replaced by *z*-scores for height or sd score for height (hSDS). Children are defined as stunted if their height-for-age is more than two sd below the WHO Child Growth Standards median (https://www.who.int/nutrition/healthygrowthproj_stunted_videos/en/). The WHO describes stunting as ‘…the result of chronic or recurrent undernutrition, usually associated with poor socioeconomic conditions, poor maternal health and nutrition, frequent illness, and/or inappropriate infant and young child feeding and care in early life’ and particularly states that ‘stunting holds children back from reaching their physical and cognitive potential’ (https://www.who.int/news-room/fact-sheets/detail/malnutrition). This may certainly be debated as it is not stunting *per* *s* *e* that holds children back from reaching their physical and cognitive potential, but rather the food shortage or their disadvantageous socio-economic status (SES) environment.

The Indonesian 2013 National Basic Health Survey^(6)^ reported a 37·2 % prevalence of stunted children under the age of 5 years. This survey also reported a 12·1 % prevalence of wasted children under the age of 5 years (below minus 2 sd from median weight-for-height of reference population), with only 2·5 % of the children being both wasted and stunted. Many Indonesian children were either short with normal weight (27·4 %) or short and overweight (6·8 %).

In order to better understand the link between the historic nomenclature of ‘percent of theoretic height and weight for age’ and modern terminology using *z*-scores, Figure 1 relates percentage of theoretic height-for-age and weight-for-age and height and weight *z*- or sd scores (based on WHO reference). Height deficits of some 15 % (85 % of theoretic height-for-age) reported by Gomez *et al*. and Pelletier correspond to height *z*-scores between −3·2 and −4·8 hSDS depending on age. These children were truly short. Assuming a usual sd of approximately 1 hSDS, these historic samples consisted of up to more than 99 % stunted children.

**Fig. 1:**
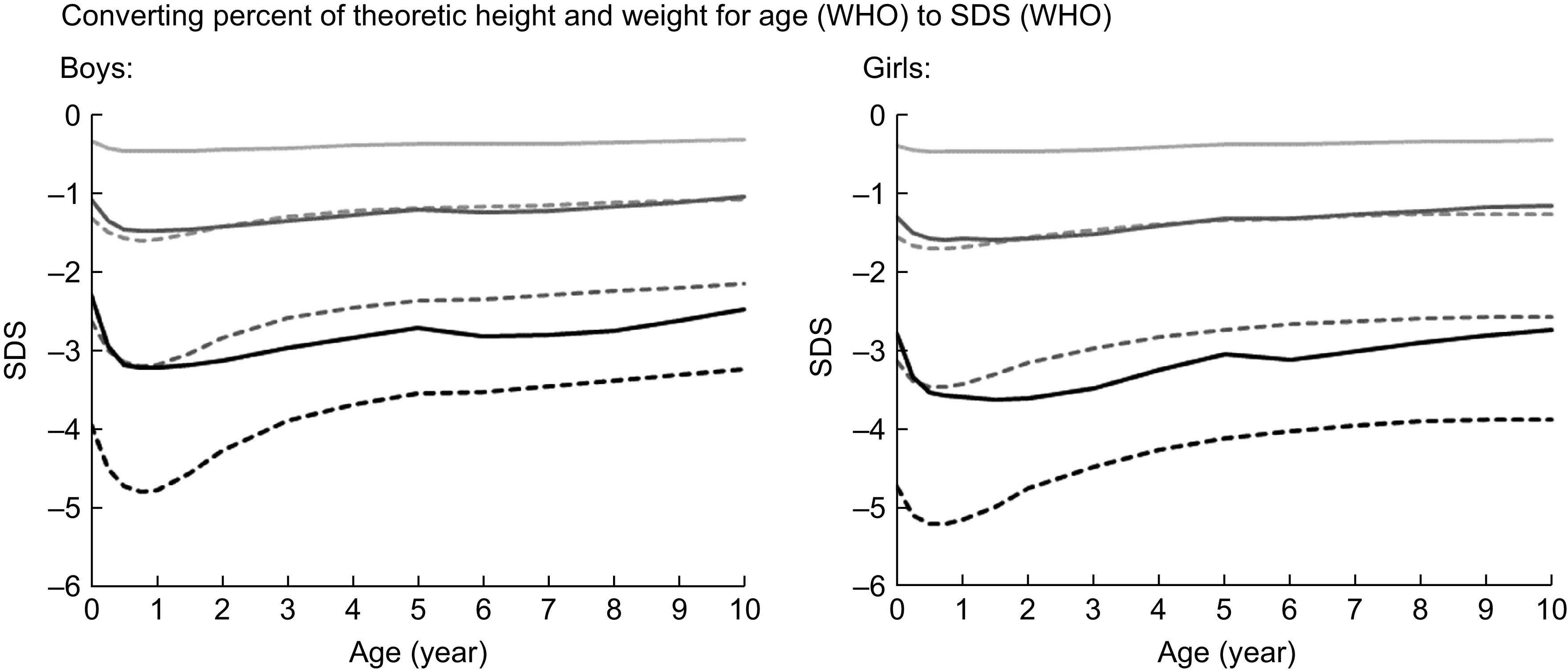
Percentage of theoretic height-for-age and weight-for-age and height and weight sd scores (based on WHO reference). 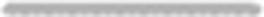, Weight 95 %; 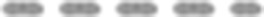, height 95 %; 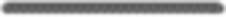, weight 85 %; 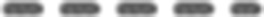, height 90 %; 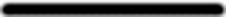, weight 70 %; 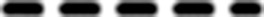, height 85 %

Children in many developing countries are short^(7–10)^. The estimate of the global prevalence of stunting for under 5-year-olds is close to 155 million^(10)^. Most published interpretations of this number interpret it to mean that the majority of these children are undernourished and in greater need for nutritional support than those whose height-for-age *z*-scores are above the critical cut-off of hSDS < 2. We do not question that all of these children are short – they are short when referred to WHO reference – but we question that all of these children are undernourished^(11)^.

Here, we describe Indonesian and Guatemalan schoolchildren with prevalence of stunting of up to 53·3 % in Indonesia and up to 56·5 % in Guatemala. These populations are short; many of these children were even below −3·2 hSDS. The question arises as to how many of these very short children are undernourished. We considered the possibility of a critical threshold (breakpoint) in the height distribution of stunted children below which body height might become a valid and reliable indicator of nutritional status. Can we distinguish between ‘mildly stunted’ children who are just short and well-fed, and ‘severely stunted’ children below some critical threshold with measurable signs of undernutrition? Considering thinness as an easily measurable sign of energetic undernutrition, we formulated the following hypothesis:

Among groups of stunted children, subgroups of very short children exist that show a relevant association between body height and thinness, defined by low BMI, low mid-upper arm circumference or low skinfold thickness.

## Samples and methods

For testing our hypothesis, we used data obtained in Indonesian and Guatemalan, two nations with a high prevalence of stunting for children <5 years old. In 2010, 35·6 % of Indonesian children were stunted and this increased to 37·2 % in 2013 (https://en.wikipedia.org/wiki/List_of_Indonesian_provinces_by_GRP_per_capita). Based on the conventional perception that stunting indicates undernutrition, Indonesian children are officially considered ‘seriously’ affected by starvation, with a Global Hunger Index of 22 (http://www.globalhungerindex.org/pdf/en/2017/posters.pdf). In contrast, Indonesia is not a poor country and ranks seventh out of 190 countries in the World Bank list of GDP (https://en.wikipedia.org/wiki/List_of_countries_by_GDP_(PPP)), which makes it difficult to understand how more than one-third of its youngest children are seriously hungry. It becomes more understandable by noting that the Global Hunger Index includes stunting as one of its four components (https://www.globalhungerindex.org/about.html). Thus, the assumption that ‘stunting = malnutrition’ is implicit.

Guatemala has a prevalence of stunting of 49·8 %, the highest in the region the Americas and the sixth highest prevalence of stunting for children <5 years old in the world. In the Maya group, the prevalence reaches almost 70 % of children <5 years old (https://www.usaid.gov/sites/default/files/documents/1864/USAID-Guatemala_NCP.pdf). Guatemala is a middle-income country and ranks 75th out of 190 countries in the World Bank list of GDP. With a global hunger index of 20·7, also Guatemala ranks within the group of nations that are considered ‘seriously’ affected by starvation (http://www.globalhungerindex.org/pdf/en/2017/posters.pdf).

Anthropometric measurements were taken from 1716 healthy Indonesian schoolchildren aged between 6·0 and 13·2 years, from three geographical regions: the urban regions Ubud (Bali) and Kupang (capital of West Timor, East Nusa Tenggara), and the in rural regions the village Soe (West Timor, East Nusa Tenggara) and Marbau (North Sumatra) in February and March 2018. The measurements were performed in the presence of the children’s teachers and supervised and accompanied by local physicians, paediatricians and medical residents^(11)^. We measured body height, weight, mid-upper arm circumference (MUAC) and triceps and subscapular skinfolds on the right site of the body. The children were lightly dressed and measured without shoes. Weight of the school uniforms was found to be close to 200 g in children below age 10 years, and about 300 g in children above age 10 years, and was subtracted from the weight measurements. Body height was determined by digital laser rangefinder GLM Professional^®^ Bosch 250 VF^(12)^ to the nearest mm, weight by digital scales (Soehnle Style Sense Compact 100) to the nearest 100 g, and skinfold thickness by caliper (Holtain, Ltd) to the nearest 0·2 mm and MUAC by non-stretchable metric tape. All measurements were taken under standardised conditions^(13)^.

Data of 3838 Guatemalan school students were obtained from the Longitudinal Study of Child and Adolescent Development conducted by the Universidad del Valle de Guatemala^(14)^. Particularly, the schoolchildren of low socio-economic status showed high prevalence of stunting. The participants were born 1963–1974, aged 4·00–18·99 years at the time of measurement and were from three SES and the two major ethnic groups of Guatemala, Ladinos and Maya. Very low SES Maya children came from a state-run primary school with grades kindergarten to sixth year, and from a private, low-cost secondary school that operated three grades. Both schools are in a village situated about 25 km from Guatemala City. The Ladino samples included an expensive private school of very high SES and a state-run school (low SES) in Guatemala City.

### Statistical analyses

Standard deviation scores for body height (hSDS) and BMI (BMI SDS) were calculated according to WHO references (https://www.who.int/growthref/en/). In the Indonesian sample, from three measures of triceps and three measures of subscapular skinfolds, the average values were calculated as final value. In the Guatemala, data set values for mean skinfold thickness (SF) were obtained from two measures of triceps and subscapular skinfolds and if within 3 mm agreement, the average of these was the final value.

**Fig. 2:**
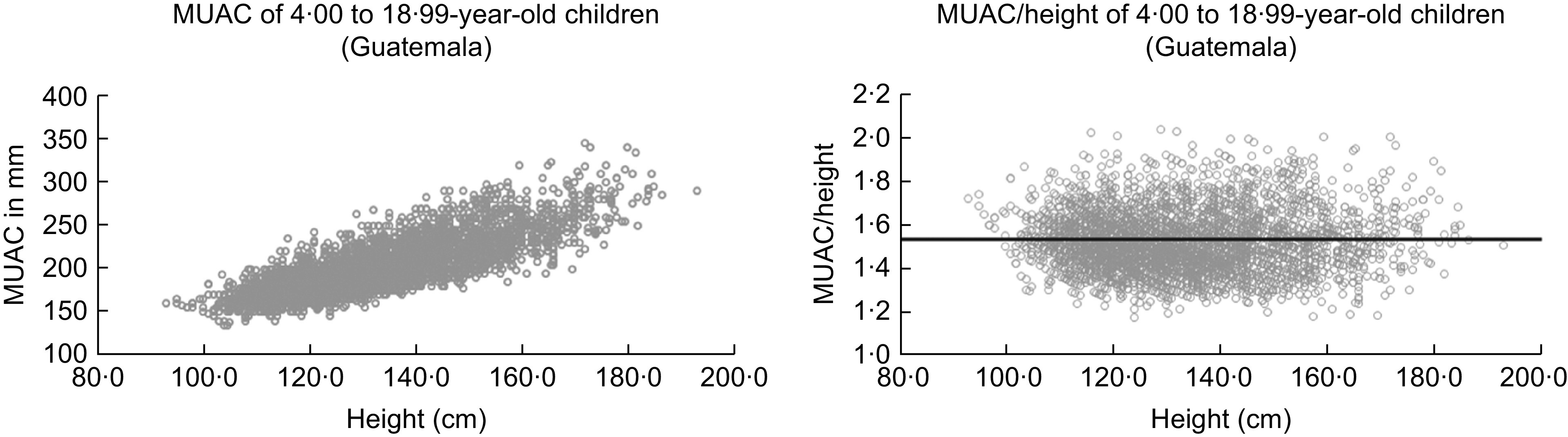
MUAC (mm) and relative MUAC to height (MUAC/h) of 4·00 to 18·99-year-old children and adolescents of our Guatemalan sample. Dark line is corresponding to the mean of MUAC/h = 1·56 by American children (after Frisancho^(15)^)

As MUAC depends on height^(15)^, we used the ratio MUAC divided by height (MUAC/h = MUAC (cm) × 10/height (cm)) for our analyses (Fig. 2). The figure illustrates the case of the Guatemalan children and shows that this ratio is almost independent of height and very similar to MUAC/h of American children^(15)^. Average MUAC/h is 1·52 (sd = 0·13) with third centile of 1·3, fifth centile of 1·32 and 10th centile of 1·36. We chose the 10th centile as a plausible cut-off according to the work of Fiorentino in Cambodian children^(16)^. Upper-arm muscle area was calculated according to Frisancho^(15)^, as Frisancho recommends this as a nutrition indicator.

**Fig. 3:**
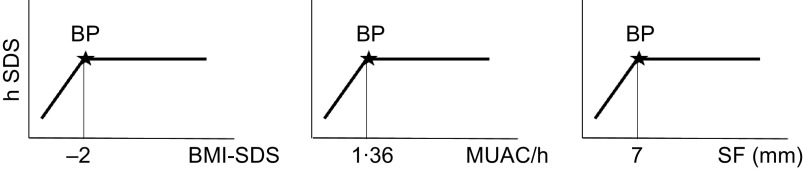
Theoretical graphs of our hypotheses, that within stunted child populations, at least subgroups might exist with measurable signs of undernutrition, that is, with BMI, mid-upper arm circumference (MUAC) and skinfold thickness (SF) below critical limits, for whom the assumption of an association between nutritional status and body height holds true. We considered the critical limit for BMI at BMI-SDS < −2 (UNICEF) the critical limit for MUAC to height at MUAC/h < 1·36 corresponding to the 10th centile of Guatemalan children (see Statistical analyses) and the critical limit for SF at SF < 7 mm corresponding to the 10th centile of well-nourished German schoolchildren (Schilitz^(18)^). BP, Breakpoint

To test our hypothesis, we searched for a threshold that might separate children who are just short, and very short children with measurable thinness for whom the assumption of a relevant association between height and thinness holds true. We used breakpoint analysis^(17)^. Breakpoint analysis segregates two segments of a linear regression. The breakpoint quantifies an abrupt change of the response function of a varying influential factor. The breakpoint can be interpreted as a critical threshold value beyond or below which a particular effect occurs. We defined the critical threshold for thinness by three criteria: (1) BMI SDS < −2, the cut-off recommended by the WHO (https://www.who.int/nutrition/healthygrowthproj_stunted_videos/en/), (2) by MUAC/h < 1·36 corresponding to the 10th centile of Guatemalan children (see above) and (3) by SF < 7 mm corresponding to the 10th centile of well-nourished German schoolchildren^(18)^. Figure 3 shows three idealised graphs illustrating associations between hSDS, with BMI SDS, MUAC/h and SF. The graphs depict no association between nutritional status and body height above, and positive associations below, the critical thresholds of thinness.

R package segmented^(17)^ was used to fit linear multiphase regressions to estimate the association between the three anthropometric indicators of the nutritional status and hSDS.

## Results

**Fig. 4:**
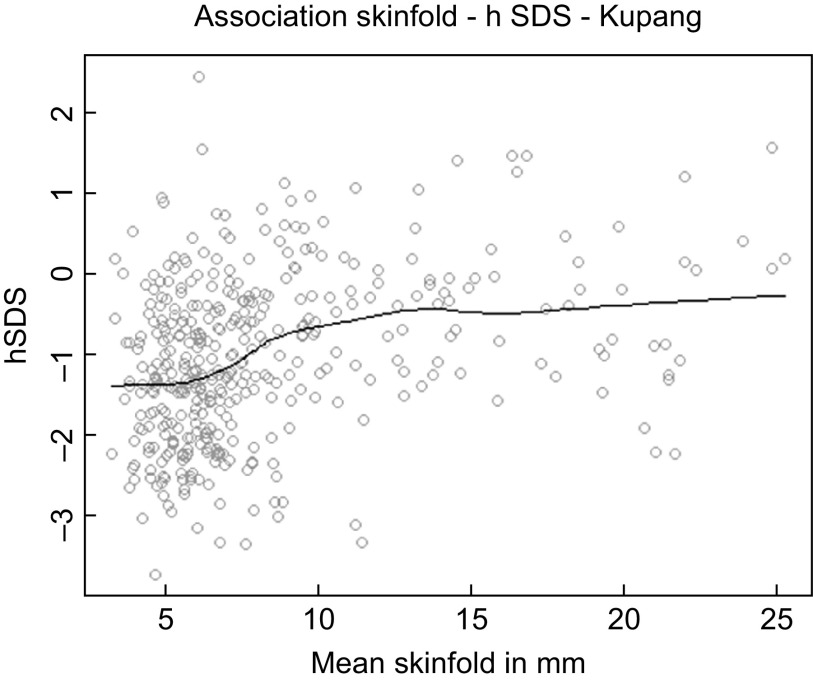
Association between mean skinfold thickness (triceps and subscapular) and height SDS (WHO reference) of children from Kupang, Indonesia. The black line represents the moving average

Figure 4 exemplifies the association between mean skinfold thickness and hSDS in the urban children from Kupang. The black line represents the Distance Weighted Least Square Regression for hSDS at different SF. The figure illustrates the prima facie impression that the thinnest children, those with an SF <~8·5 mm, are shorter.

Yet, this impression is deceptive. The breakpoint analysis distinguished between Kupang children with skinfold thickness above and below 10·16 mm (Table 1). This threshold is significantly larger than the critical threshold for thinness at SF < 7 mm and close to the 50th centile of healthy German children^(18)^. It indicates that the majority of these Indonesian children cannot be undernourished, at least by this criterion.

**Table 1:** Breakpoint (BP) analyses of the association between BMI SDS (WHO reference), upper arm circumference related to height (MUAC/h) and mean skinfold thickness (SF; mean of triceps and subscapular skinfold) and height-SDS (hSDS) (WHO reference) of children from Indonesia *(Ubud, Marbau, Kupang, Soe) and Guatemala (high SES, low SES Ladino, low SES Maya)*†

	*β*	se	*P*	BP	se	*R* ^2^
BMI SDS						
Indonesia						
Ubud (*n* 591)						
(Intercept)	−0·42	0·04	<2e−16***			
BMI SDS_1	0·30	0·03	**<2e**−16***	**4·45**	0·07	0·198
BMI SDS_2	−13·70	5·49	NA			
Marbau (*n* 791)				no breakpoint detected		
Kupang (*n* 402)						
(Intercept)	−1·81	0·52	0·000***			
BMI SDS_1	−0·10	0·17	0·54	−2·27	0·48	0·172
BMI SDS_2	0·40	0·17	NA			
Soe (*n* 220)						
(Intercept)	−1·84	0·17	<2e−16***			
BMI SDS_1	0·15	0·09	0·1	−0·31	0·57	0·082
BMI SDS_2	0·53	0·28	NA			
Guatemala						
High SES (*n* 1604)						
(Intercept)	−1·09	0·35	0·001**			
BMI SDS_1	−0·38	0·20	0·065	−1·23	0·22	0·052
BMI SDS_2	0·64	0·21	NA			
Low SES Ladino (*n* 1269)						
(Intercept)	−1·74	0·03	<2e−16***			
BMI SDS_1	0·14	0·04	0·000***	**+1·10**	0·18	0·068
BMI SDS_2	0·82	0·21	NA			
Low SES Maya (*n* 1111)						
(Intercept)	−5·34	2·5	0·034*			
BMI SDS_1	−1·4	1·03	0·175	−1·95	0·36	0·04
BMI SDS_2	1·66	1·03	NA			
MUAC/h						
Indonesia						
Ubud						
(Intercept)	1·04	1·61	0·52			
MUAC/h_1	−11·24	12·03	0·35	0·142	0·006	0·068
MUAC/h_2	24·77	12·24	NA			
Marbau						
(Intercept)	−0·57	0·77	0·46			
MUAC/h_1	−5·33	5·08	0·30	0·166	0·005	0·053
MUAC/h_2	34·44	8·46	NA			
Kupang						
(Intercept)	0·43	0·90	0·64			
MUAC/h_1	−6·6	7·50	0·38	0·129	0·007	0·060
MUAC/h_2	20·55	8·08	NA			
Soe						
(Intercept)	0·87	1·80	0·63			
MUAC/h_1	−22·01	13·82	0·11	0·139	0·005	0·025
MUAC/h_2	45·65	19·55	NA			
Guatemala						
high SES						
(Intercept)	19·24	8·68	0·027*			
MUAC/h_1	−15·21	6·99	0·030*	1·279	0·024	0·007
MUAC/h_2	15·07	6·99	NA			
Low SES Ladino						
(Intercept)	0·42	0·49	0·387			
MUAC/h_1	−1·44	0·33	1·6e−05***	1·635	0·026	0·025
MUAC/h_2	4·71	0·95	NA			
Low SES Maya						
(Intercept)	0·88	2·53	0·729			
MUAC/h_1	−2·17	1·92	0·260	1·361	0·069	0·007
MUAC/h_2	1·68	1·95	NA			
SF (mm)						
Indonesia						
Ubud						
(Intercept)	−1·55	0·2	1·64e−14***			
SF_1	0·12	0·02	**2·22e**−07***	**12·23**	1·41	0·149
x̄SF_2	−0·10	0·03	NA			
Marbau						
(Intercept)	6·04	3·72	0·105			
SF_1	−1·71	0·93	0·066	4·47	0·33	0·082
SF_2	1·77	0·93	NA			
Kupang						
(Intercept)	−2·09	0·22	<2e−16***			
SF_1	0·14	0·03	**2·89e**−5***	**10·16**	1·99	0·112
SF_2	−0·11	0·04	NA			
Soe						
(Intercept)	−1·47	1·08	0·174			
SF_1	−0·13	0·23	0·569	5·26	0·84	0·035
SF_2	0·27	0·24	NA			
Guatemala						
High SES						
(Intercept)	−0·97	0·23	2·13e−05***			
SF_1	0·1	0·0$	0·00784**	**8·00**	1·04	0·017
SF_2	−0·07	0·04	NA			
Low SES Ladino						
(Intercept)	−3·94	1·13	0·000***			
SF_1	0·51	0·31	0·096	4·06	0·38	0·038
SF_2	−0·09	0·04	NA			
Low SES Maya						
(Intercept)	−2·49	0·08	<2e−16***			
SF_1	0·05	0·01	**3·56e**−06***	**13·51**	3·20	0·026
SF_2	−0·07	0·04	NA			

NA, not available.

Significance: * low, ** middle, *** high.

† Values in the column BP indicate the localisation of the BP; *β* indicates the slope of the linear regression lines before (*_1) and after the BP (*_2). Bold numbers indicate significant BP separating associations between indicators of nutritional status and hSDS below and above the BP. *R*^2^ refers to the entire model.

For BMI, we either found no breakpoints (Marbau) or breakpoints that failed to reach statistical significance (Kupang, Soe and Guatemala in high SES Ladinos and low SES Maya, respectively). We found a significant breakpoint in the schoolchildren of Ubud and Guatemalan low SES Ladinos. Yet, the breakpoints were above the critical threshold of thinness (BMI SDS < −2), at +4·45 and +1·10 BMI SDS, respectively. Also the analysis of MUAC/h failed to identify stunted individuals. Breakpoints were detected in the children of Guatemala (high SES and low SES Ladino), but suggested negative associations between mid-upper arm circumference and height, that is, below the breakpoint, the shorter individuals had larger MUAC. In other words, neither BMI SDS nor mid-upper arm circumference supported the hypothesis of height growth impairment in exceptionally thin children. There were no breakpoints in the association between upper arm muscle area^(15)^ and hSDS (results not shown in detail). In the case of skinfold thickness, we similarly failed to identify stunted individuals. We detect statistically significant breakpoints in the schoolchildren of urban Ubud, Kupang and high SES as well low SES Maya-children of Guatemala. Yet, none of these breakpoints appeared plausible from a nutrition and public health perspective. It was found that Ubud, Kupang and low SES Maya-children increased in height with increasing skinfold thickness, but this trend occurred well above the threshold of thinness. The trend likely reflects the general stimulation of overweight and obesity on height^(19)^.

In order to further evaluate the putative association between nutritional status and height, we determined the coefficients of regression (*R*^2^) below all significant breakpoints for each row of Table 1 separately. Yet, the coefficients remained statistically insignificant or had negligible effect size (Ubud – BMI SDS: *P* < 0·001, *R*^2^ = 0·064; x̄SF: *P* = 0·74, *R*^2^ = 0·0003; Kupang – x̄SF: *P* = 0·431, *R*^2^ = 0·0019; high SES – x̄SF: *P* = 0·743, *R*^2^ = 0·0001; low SES Maya – x̄SF: *P* < 0·001, *R*^2^ = 0·023; low SES Ladino – BMI SDS: *P* = 0·737, *R*^2^ = 0·009). The *R*^2^ for x̄SF with height of the high SES Guatemalan children at 0·017 showed that any putative association between indicators of nutritional status and height was negligible.

We considered that chronological age of the participants might influence the prevalence of stunting. One reviewer of this articles suggested that participants <10 years old might be more stunted than older participants. We found that the percentage of stunted children did not vary with age. The association between height SDS and age was negligible with *r* = 0·12 (*P* < 0·01) in the Indonesian children, and insignificant in the Guatemalan children.

## Discussion

The breakpoint analysis failed to support our hypothesis that subgroups of very short children and youth exist with a relevant association between thinness and body height. There is no apparent relation between BMI, MUAC and skinfold thickness as indicators of the nutritional status, and body height in the Indonesia and Guatemala samples, both with high prevalence of stunting.

It is not new and has never been questioned that undernutrition is terrible and impairs growth. Yet, short stature on its own is neither a synonym of undernutrition^(11)^ nor an appropriate indicator of undernutrition. Already in 1916, the German pediatrician Meinhard von Pfaundler^(20)^ summarised body mass studies in children before World War I. Even though he considered food as a potentially influencing factor, he stated: ‘that the undernourishment of the children of the poor, with the exception of the fact that it certainly does not occur in the assumed extent, is probably over-estimated in its importance for the growth of body length’. Shortly after World War I, Schlesinger^(21)^ wrote: ‘The child’s longitudinal growth is largely independent of the extent and nature of the diet… Even during severe dietary restrictions, impairments of infant growth are markedly small, and occur slowly and delayed. Only during severe infectious nutritional disorders of the infant… Stolte and others^(22)^ observed a temporary growth inhibition… Malnourished infants show an inhibition of longitudinal growth only, and especially during periods of reparation, when food supplies, e.g. breast milk, was low in protein and minerals, but they quickly recovered when given protein rich milk’. Screening for undernutrition has always been an important issue. After World War I, the American Quaker Children’s Aid Mission offered additional meals to undernourished German schoolchildren and requested from the German paediatricians to better define undernutrition and to develop criteria for classifying the degree of undernutrition of a given child.

This was not a trivial task. Simply taking height and weight was considered inappropriate, and instead the paediatricians discussed indices. Pfaundler^(20)^ originally introduced Livi’s (ponderal) index. Livi’s index – 100 (√3 weight)/body height) – relates the cube root of weight to height and was considered to better mirror the nutritional state than body weight alone, with arguments similar to those used today to recommend the BMI. Later, Pfaundler suggested an index that had recently been introduced by Pirquet. The index ignored leg length and thus appeared to better mirror the nutritional status. This ‘Pelidisi’ index – Pondus dEcies LInear DIviso SedentIs altitudo = 100 (√3 weight)/sitting height) – was then thoroughly discussed and appreciated by Wagner in his work on the numerical assessment of the nutritional status in 1921^(23)^. He discussed practical aspects of the necessity to determine the nutritional status of children for detecting the poor, and those who are in need of ‘warm food’. Wagner discussed possibilities to better define a state of undernutrition. He wrote that ‘…the precondition for the usability of a body fullness index is that it represents an unnamed ratio derived from the division of equidimensional quantities’. He rejected indices such as weight-for-height because they divide a three-dimensional size, the weight, by a one-dimensional length, which results in an area. Yet, in spite of these thoughts, the pelidisi was never widely accepted and eventually disappeared from the literature presumably due to arithmetic clumsiness at a time when computers did not exist. In 2015, Burton^(24)^ presented similar thoughts recommending that BMI be replaced by an index of body build that is less dependent on relative leg length and age in children and adults than are the BMI and the Rohrer Index and proposed Weight/Sitting Height^3^.

Half a century after Pirquet, Pfaundler and Wagner, and apparently unswayed by their considerations, Jelliffe^(25)^ introduced mid-upper arm circumference (in those days called arm girth) as a new practical, useful and sensitive index for assessing protein–energy malnutrition (PEM) in early childhood. The rationale was that the arm girth depicts a heavily muscled area with good quantity of fat. In PEM, all musculature is presumed to be uniformly affected^(26)^. McKay^(27)^ reported positive correlation between arm girth and weight in Malaysian children in 1969. In contrast to this historic observation, the stunted children of modern Indonesia and Guatemala appear well-nourished, some of them significantly obese, and thus lack convincing evidence of undernutrition. We failed to detect meaningful associations between weight, BMI, skinfold thickness and mid-upper arm circumference with body height in these children. Even using the more elaborate statistical tool of breakpoint analyses, we failed to detect subpopulations among these children for whom the common perception of an existing association between thinness and short stature might hold true.

Meanwhile, the conventional wisdom of nutritional epidemiology seems to change gradually. A slowly increasing number of articles in the current literature start to question if stunting is the single indicator to reliably detect poor child nutrition. In 2014, Becker *et al*.^(28)^ and later Bouma^(29)^ stated that cut-offs at −2 height SDS and −1 height SDS for diagnosing moderate and mild undernutrition are not useful and should be replaced either by combinations of indicators, such as loss in lean body mass, infections, delayed wound healing and others.

Undernutrition needs intervention. But, how to define undernutrition by anthropometry? At the beginning of the 20th century, it was obvious to the paediatric community that neither height nor weight contained that specific information. The indices of Quetelet, Livi and Pelidisi were soon dismissed. Re-inventing stunting as a measure of nutrition deficit in the later part of the 20th century was neither new nor original and is showing signs of its shortcomings. It appears that better indicators for undernutrition than some trivial SDS of < −2·0 sd in height are needed. Systematic reviews show that existing anthropometric cut-offs for detecting nutrition deficits and for planning nutrition interventions have little to no public health impact^(30–32)^.

It is well-known that emotional deprivation and psychological stress can cause growth failure and short stature. These non-nutritional causes of stunting are known as ‘hospitalism’^(33)^ and psychosocial short stature^(34,35)^ and are associated with impaired growth hormone secretion. Growth failure due to emotional deprivation may be followed by catch-up after appropriate intervention and treatment as described more than half a century ago, by Widdowson, Talbot *et al*. and Bakwin^(36–38)^.

Building on these fundamental clinical observation, we have proposed that much of the current short stature/stunting observed in infants and children from impoverished families of low- and middle-income countries has causes due more to emotional/psychological stress and insecurity rather than undernutrition *per* *s**e*^(39–41)^.

A relatively large study of growth failure related to emotional factors was published more than a century ago. Igl^(42)^ published rich data on catch-up growth in height of 4896 school boys and 4612 school girls from a boarding school from Brno, school year 1903/1904. He compared height increments during 10 months of school attendance with increments during interposed 2 months of summer holiday (Table 2). He documented remarkable catch-up in height with up to 3·5 cm during holiday, corresponding to a height velocity of up to 21 cm/year. These growth rates by far outranged average growth of schoolchildren at that time and surpassed the growth rate during the school term by almost a factor of six. As urban Austrian school children before World War I have neither been reported chronically undernourished, nor chronically ill, we interpret the annual school-related period of growth depression followed by catch-up as an expression of significant familial emotional deprivation temporarily interrupted by emotional support during summer holidays.

**Table 2:** Height increments of children of a boarding school, Brno, school year 1903/1904, during 10 months of school attendance and during interposed 2 months of summer holiday at the end of each school year*

Age (years)	7	8	9	10	11	12
Boys						
*n*	834	804	870	916	798	674
School year (10 months)	2·5	2·8	2·4	2·3	2·4	2·8
Height velocity (cm/years)	3·00	3·36	2·88	2·76	2·88	3·36
Holiday (2 months)	2·9	2·2	2·9	1·4	1·9	3
Height velocity (cm/years)	17·40	13·20	17·40	8·40	11·40	18·00
Girls						
*n*	854	754	770	808	728	698
School year (10 months)	2·4	2·6	2·9	2·4	2·7	3·4
Height velocity (cm/years)	2·88	3·12	3·48	2·88	3·24	4·08
Holiday (2 months)	3·1	3·5	1·2	1·9	3·5	0·6
Height velocity (cm/years)	18·60	21·00	7·20	11·40	21·00	3·60

* Catch-up in height with up to 3·5 cm during holiday, corresponding to a height velocity of up to 21 cm/year, by far outranged growth velocity during the 10 months of school attendance.

### A way forward in nutritional epidemiology

We suggest changing the focus of nutritional epidemiology. Anthropometric variables and ratios of these variables are stationary descriptions of a particular condition. Such variables provide a momentary glance. We instead suggest taking a more dynamic view, focusing on responsiveness. Catch-up growth is well known and occurs when successfully recovering from food shortage, illness or other forms of developmental impairment. Children catch-up both in weight and in height. Catch-up growth is cause-specific. It follows periods of growth inhibition, and it is characterised by weight and height velocity above the limits of normal for age^(43)^.

Studies published shortly after World War I showed catch-up growth of 3–5 cm in height within 6–8 weeks on re-feeding, in starved German children after being transferred to care families or communal lodges in Switzerland^(30,31)^. Catch-up growth after re-feeding and after recovering from major illness has later extensively been presented in the clinical paper of Prader *et al*.^(44)^. Catch-up growth occurs in migrant children when moved into more prosperous conditions^(14)^. In 1994, Golden^(45)^ reviewed the possibilities of catch-up growth in stunted malnourished children and concluded that ‘it seems that most malnourished children retain their capacity for full catch-up’.

Catch-up growth is an immediate and sensitive indicator of a variety of both biological and psychosocial factors influencing child growth. A catch-up growth spurt may follow improvements of previous growth impairment within a few days and manifests itself by either one single large or serial mini growth spurts^(45)^. Catch-up growth characterises the dynamic physical response to preceding developmental malfunction. Using this approach for diagnosing developmental impairment fundamentally differs from any stationary measure of anthropometric variables and ratios.

Many modern nutrition interventions in the stunted child populations of low- and middle-income countries lack relevant catch-up in height and thus strongly suggest that it is not nutrition that keeps these children short^(32,46)^. We consider education and social-economic-political-emotional factors being most significantly involved in the high prevalence of child stunting in these countries. More and better-quality education and a higher quality of a broad range of social-economic-political-emotional factors have long been associated with better maternal health, higher birth weight, healthier physical growth, more successful schooling and greater earnings in adult employment^(39–41,46)^. In view of the ease to perform repetitive daily or short-term (e.g. 4–6 weeks) measurements of height and weight in children^(47)^, we suggest to abstain from single measures of height, weight and related ratios and instead utilise the dynamic physical response of length/height catch-up to preceding developmental malfunction as a more appropriate indicator of successful growth promotion in the individual short child.

In summary, the catch-up growth spurt following a nutrition intervention is a better indicator of undernutrition than static thresholds for stunting. Absence of catch-up growth following re-feeding strongly indicates non-nutritional causes of stunting.

## Footnotes

* The term ‘malnutrition’ was traditionally used to describe undernutrition, but with the onset of the obesity epidemic it has been extended to describe both the lack of essential nutrients and the excess. The term ‘undernutrition’ refers specifically to the lack of essential nutrients.
